# Hemodynamic effects of volume expansion on left ventricular-arterial interactions in circulatory shock—a prospective observational study

**DOI:** 10.3389/fcvm.2026.1726410

**Published:** 2026-01-27

**Authors:** Xiaoyang Zhou, Hanyuan Fang, Tao Pan, Chang Xu, Jianneng Pan, Bixin Chen, Zhaojun Xu, Caibao Hu

**Affiliations:** 1 Department of Intensive Care Medicine, Ningbo No. 2 Hospital, Ningbo, Zhejiang, China; 2 Department of Emergency, Ningbo Yinzhou No. 2 Hospital, Ningbo, Zhejiang, China; 3 Department of Intensive Care Medicine, Affiliated Zhejiang Hospital, Zhejiang University School of Medicine, Hangzhou, Zhejiang, China

**Keywords:** cardiovascular physiology, circulatory shock, fluid responsiveness, hemodynamics, ventricular-arterial coupling, ventricular-arterial interactions, volume expansion

## Abstract

**Introduction:**

Left ventricular-arterial interaction, also termed ventricular-arterial coupling (VAC), determines cardiovascular efficiency by matching cardiac performance and arterial functions, and comprehensively assesses cardiac contractility and arterial load in a common framework. Volume expansion is a commonly used hemodynamic measure in the management of circulatory shock. However, its hemodynamic effects on each component of the cardiovascular system are not fully understood. This study aimed to evaluate the effects of volume expansion on the left VAC and determine whether the left VAC is associated with fluid responsiveness in circulatory shock.

**Materials and methods:**

This prospective observational study enrolled mechanically ventilated patients with circulatory shock, for whom the attending physician decided to perform a fluid challenge. Hemodynamics and left VAC were measured immediately before and after the fluid challenge. Fluid responsiveness was defined as a 15% or greater increase in cardiac index following the fluid challenge. Left VAC was quantified by the ratio of arterial elastance (Ea) to left ventricular end-systolic elastance (Ees), measured by echocardiography. Multivariable logistic regression analyses were used to identify hemodynamic variables associated with fluid responsiveness.

**Results:**

Of 58 enrolled patients, 26 (44.8%) were fluid responders. Fluid responders exhibited a higher baseline Ea/Ees ratio compared to non-responders (1.22 ± 0.28 vs. 1.02 ± 0.30, *P* = 0.011), while the Ea and Ees were comparable between them. Volume expansion caused significant reductions in the Ea and the Ea/Ees ratio in fluid responders, and led to remarkable increases in the Ea and the Ea/Ees ratio in non-responders, while the Ees remained unchanged in both groups. The baseline Ea/Ees ratio was significantly correlated with the fluid-induced changes in cardiac index (*r* = 0.373, *P* = 0.004). Multivariable regression analysis suggested that the baseline Ea/Ees ratio was independently associated with fluid responsiveness after adjusting for confounders (odds ratio 1.339, 95% confidence interval: 1.075–1.668, *P* = 0.009).

**Conclusion:**

In mechanically ventilated patients with circulatory shock, volume expansion optimized the left VAC in preload-dependent patients, primarily by reducing arterial load, and the baseline left VAC was associated with fluid responsiveness.

## Introduction

Volume expansion (VE) is a commonly used hemodynamic measure in the management of circulatory shock. The efficacy of VE depends on whether the heart operates on the steep portion of the Frank-Starling curve, known as fluid responsiveness (1). The prediction of volume responsiveness has been studied for decades, and numerous tests and dynamic indices have been developed and validated (1). Although these indices are accurate for predicting preload dependence, they seem unable to provide additional information about the VE-associated cardiovascular physiological changes. Indeed, VE may induce varied changes in each component of the cardiovascular system in patients with different cardiovascular profiles, resulting in diverse hemodynamic responses (2, 3). Therefore, understanding the cardiovascular physiology involved during VE is an essential procedure supplementary to predicting preload dependence.

In the cardiovascular system, left ventricular-arterial interaction, also termed ventricular-arterial coupling (VAC), refers to the interdependent and interactive relationship between the heart and its downstream vascular network, measured by the ratio of effective arterial elastance (Ea) to left ventricular end-systolic elastance (Ees) (4, 5). Therefore, left VAC can offer insights into the adequacy of one component of the cardiovascular system relative to the other, and thus reflects the status of cardiovascular equilibrium (6–8). Left VAC determines cardiovascular efficiency and energetics by matching cardiac performance and arterial functions, aiming to deliver optimal cardiac output (CO) to meet metabolic demand (4–9). In other words, ventricular-arterial interactions (i.e., left VAC) determine cardiac ejection and arterial pressure given a specific cardiac preload. Theoretically, any hemodynamic intervention (including VE) affecting these components will potentially alter ventricular-arterial interactions, and a mismatched ventricular-arterial interaction may lead to diminished CO, even at an optimal volume status (10). This underlies a potential theoretical rationale for the relationship between left VAC and fluid responsiveness. Furthermore, assessing left VAC may allow for a more comprehensive understanding of the VE-induced physiological changes concerning each component of the cardiovascular system. Previously, a clinical study with a small sample size has revealed the relevance of left VAC and fluid responsiveness in patients undergoing cardiothoracic surgery (11). However, the hemodynamic effects of VE on each component of the cardiovascular system are not fully understood. Therefore, we conducted this study to assess the hemodynamic effects of VE on left ventricular-arterial interactions and evaluate the association between left VAC and fluid responsiveness in patients with circulatory shock.

## Materials and methods

This was a prospective observational study conducted in the intensive care unit (ICU) of Ningbo No.2 Hospital, China, from January 2024 to December 2024. We obtained the written informed consent for each patient from their relatives. As part of a study protocol registered at the Chinese Clinical Trial Registry (ChiCTR2100053665), this study was approved by the institutional ethics committee of Ningbo No. 2 Hospital (YJ-NBEY-KY-2022-147-01), and the manuscript was drafted following the STROBE guidelines (12). This study was conducted in compliance with the Declaration of Helsinki.

### Study subjects

The eligible study subjects were mechanically ventilated adult patients (aged 18 years or older) with circulatory shock, for whom the attending physician decided to perform a fluid challenge. The diagnosis of circulatory shock was considered if one or more of the following signs were present: 1) systolic arterial pressure (SAP) <90 mmHg, mean arterial pressure (MAP) <65 mmHg, or need for vasopressors; 2) skin mottling; 3) urine output <0.5 mL/kg/h for 2 or more hours; 4) lactate level >2 mmol/L (13). Fluid challenge was indicated in the presence of arterial hypotension (SAP <90 mmHg or MAP <65 mmHg) or clinical signs of hypoperfusion (skin mottling, oliguria, lactate level >2 mmol/L). However, patients who met any of the following criteria would be excluded: 1) absence of indwelling arterial or central venous catheterization; 2) valvular heart disease; 3) use of extracorporeal membrane oxygenation, intra-aortic balloon pump, or pacemaker; 4) contraindications to VE; 5) poor echogenicity, or contraindications or intolerance to transthoracic echocardiography (TTE); 6) onset of atrial fibrillation during the study; 7) refractory shock expected to result in death within 24 h; 8) decline to participate.

### Study protocol

All enrolled patients received standard treatment for circulatory shock (13, 14). To prevent spontaneous breathing efforts, sedative and analgesic drugs were administered continuously, and a pressure-controlled ventilation mode was used for all patients studied. As part of routine procedures, invasive radial artery and central venous catheterizations were performed, and an electrocardiogram and pulse oximetry were continuously monitored. Pressure calibration was performed in the supine position, with transducers zeroed at the midpoint of the fourth intercostal space at the midaxillary line (the right atrium's level) (15). After deciding to perform the fluid challenge test, we recorded hemodynamic variables and performed a TTE to measure left VAC before the fluid challenge, with the patients lying in the 45° semi-recumbent position. Then, a pressurized fluid bolus of 500 mL of Ringer's solution was administered over 15 min to conduct the fluid challenge. A second set of measurements, similar to the initial ones, was recorded immediately after the test. Notably, we did not modify the ventilator settings, vasopressor doses, or doses of sedative and analgesic drugs during the fluid challenge.

### Data collection

Demographic characteristics, comorbidities, and causes of circulatory shock were recorded at enrollment. We also calculated the Acute Physiology and Chronic Health Evaluation II score and Sequential Organ Failure Assessment (SOFA) score on the day of enrollment. Additionally, we documented ventilator parameters, sedative and analgesic drugs, and vasoactive agents used during the study period. Arterial lactate levels and central venous oxygen saturation were measured before VE. Hemodynamic variables and ultrasound parameters were assessed immediately before and after the fluid challenge. ICU mortality and ICU stay duration were also recorded.

### Transthoracic echocardiography examination

TTE examination was performed immediately before and after the fluid challenge, regardless of the respiratory cycle, by an independent ICU physician using the CX50 ultrasound system (Philips Medical System, Suresnes, France). The operator had extensive operational experience and was blinded to the study outcomes. Left ventricular ejection fraction (LVEF) was measured on the apical four-chamber view using Simpson's method, and stroke volume (SV) was calculated with the formula SV = VTI × LVOT area, where VTI is the aortic velocity-time integral, and LVOT refers to the left ventricular outflow tract. Consequently, indexed SV (SVI) was computed as SV divided by body surface area, and cardiac index was calculated as SVI multiplied by heart rate (HR). On the apical five-chamber view, VTI, pre-ejection time (T_pre−e_), and total systolic time (T_tot−s_) were measured simultaneously using continuous Doppler transaortic flow and electrocardiogram, with T_pre−e_ from R-wave to flow onset and T_tot−s_ from R-wave to end-flow. The average of three consecutive measurements was used as the representative value for each parameter. Finally, the Ea was equal to ESP/SV, where ESP represents left ventricular end-systolic pressure and is estimated by 0.9 × SAP, and the Ees was calculated by using the single-beat method, as previously described (7). Therefore, the left VAC was measured by the ratio of Ea to the Ees (i.e., Ea/Ees ratio). Additionally, we estimated the systemic vascular resistance index (SVRI) as (MAP—CVP)/cardiac index and arterial compliance (C_A_) as SV divided by pulse pressure, where CVP is the central venous pressure.

### Definition

The fluid-induced absolute changes in each hemodynamic variable (Δ) were calculated by subtracting the baseline value from the value after VE. However, to facilitate defining fluid responsiveness, the change in cardiac index was calculated as the relative change: (the value after VE—the baseline value)/the baseline value ×100%. We predefined fluid responsiveness as at least a 15% increase in cardiac index induced by VE.

### Statistical analysis

SPSS version 26.0 (IBM, New York, USA) was used to perform statistical analyses. We assessed the normal distribution for each continuous variable using the Kolmogorov–Smirnov test. Continuous data were presented as means ± standard deviation (SD) or medians with interquartile ranges (IQR), depending on the data distribution, while categorical variables were reported as frequencies (percentages). Inter-group comparisons of continuous data were conducted using the Student's *t*-test or the Mann–Whitney test, as appropriate. Intra-group comparisons employed the Student's paired *t*-test, and categorical variables were compared using the Chi-squared test or Fisher's exact test, as appropriate.

Relationships between variables were evaluated using the Pearson correlation coefficient (r). The univariate and multivariate logistic regression models were used to identify hemodynamic variables associated with fluid responsiveness, and variables with a *P-*value of <0.1 in the univariate model were included in the multivariate model using a step-wise backward likelihood ratio method. The multicollinearity was eliminated by correlation check and the variance inflation factor (VIF) method. The area under a receiver operating characteristic curve (AUC) was determined to assess the diagnostic accuracy, using MedCalc Statistical Software (MedCalc Software bvba, Ostend, Belgium). The optimal cutoff value was determined based on the maximum Youden index. To assess intra-operator reproducibility for TTE, we randomly selected 15 patients from the studied cohort to compute the coefficient of variation and the least significant change. A sample size of 50 subjects provided sufficient statistical power to test the hypothesis that the Ea/Ees ratio predicts fluid responsiveness, assuming an AUC of 0.75, an alpha risk of 0.05, and a beta risk of less than 0.1. A two-tailed *P*-value < 0.05 was considered statistically significant.

## Results

**Figure 1:**
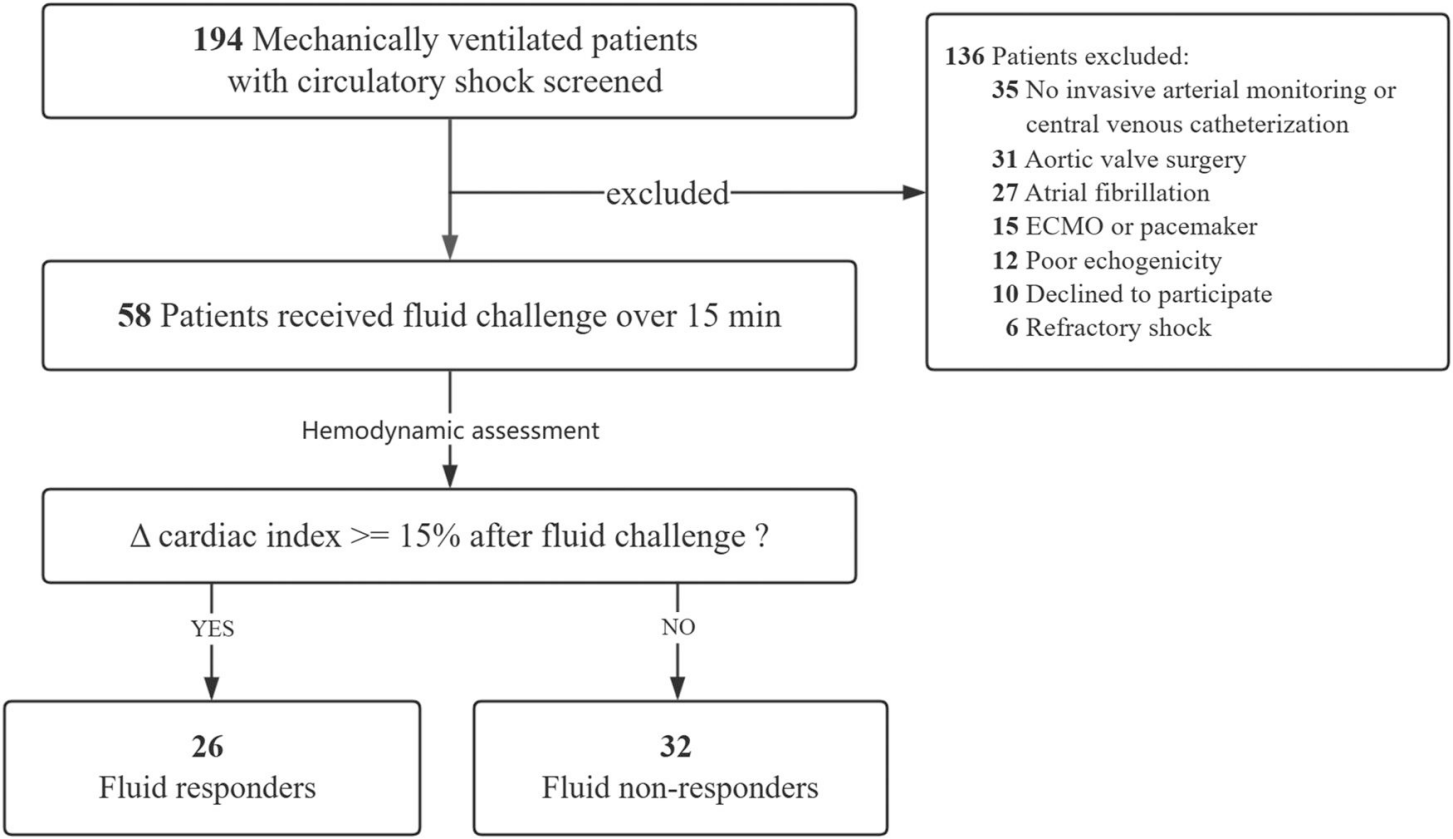
Flowchart of patient selection. ECMO extracorporeal membrane oxygenation.

As shown in Figure 1, a total of 58 patients met the selection criteria and were consecutively included in this study. Among them, 26 (44.8%) patients were fluid responders. The demographic and clinical characteristics were similar between fluid responders and non-responders (Table 1). Of note, fluid responders had a significantly lower SOFA score at enrollment than non-responders; one patient did not receive norepinephrine infusion before VE. The intra-operator reproducibility for TTE was considered acceptable (see Supplementary Table S1).

**Table 1:** Demographic and clinical characteristics.

Variables	Fluid responders (*n* = 26)	Fluid non-responders (*n* = 32)	*P*-value
Age (years), median (IQR)	71 (54, 77)	73 (62, 78)	0.628
Male/female, *n*	15/11	21/11	0.536
Body mass index (kg/m^2^), mean ± SD	22.3 ± 3.9	23.9 ± 3.5	0.114
Comorbidities, *n* (%)			
Hypertension	14 (53.8)	20 (62.5)	0.506
Diabetes	7 (26.9)	7 (21.9)	0.655
Coronary heart disease	5 (19.2)	6 (18.8)	0.963
Chronic kidney disease	2 (7.7)	3 (9.4)	0.820
Etiologies of shock, *n* (%)			
Distributive	20 (76.9)	26 (81.3)	0.775
Hypovolemic	4 (15.4)	3 (9.4)	
Cardiogenic	2 (7.7)	3 (9.4)	
APACHE II score, mean ± SD	18 ± 6	20 ± 5	0.166
SOFA score, mean ± SD	8 ± 3	10 ± 3	0.016
Arterial lactate (mmol/L), median (IQR)	1.3 (0.9, 2.6)	1.7 (1.1, 2.5)	0.425
ScvO_2_ (%), median (IQR)	65 ± 14	68 ± 15	0.453
Tidal volume (mL/kg of PBW), mean ± SD	8.0 ± 2.0	7.8 ± 1.2	0.656
Driving pressure (cmH_2_O), median (IQR)	12 (10, 13)	13 (10, 14)	0.450
PEEP (cmH_2_O), median (IQR)	5 (5, 8)	5 (5, 8)	0.458
Analgesia and sedation, *n* (%)			
Midazolam	16 (61.5)	24 (75.0)	0.270
Propofol	10 (38.5)	9 (28.1)	0.404
Fentanyl	7 (26.9)	11 (34.4)	0.542
Butorphanol	7 (26.9)	10 (31.3)	0.719
Duration of invasive mechanical ventilation (days), median (IQR)	13 (6, 19)	8 (3, 17)	0.250
Dose of norepinephrine (μg/kg/min), median (IQR)	0.17 (0.10, 0.34)	0.23 (0.18, 0.29) (*n* = 31)	0.294
Inotropic agents, *n* (%)	8 (30.8)	11 (34.4)	0.771
Length of ICU stay (days), median (IQR)	13 (8, 24)	11 (4, 17)	0.225
ICU mortality, *n* (%)	5 (19.2)	8 (25.0)	0.600

SD, standard deviation; IQR, interquartile range; APACHE, acute physiology and chronic health evaluation; SOFA, sequential organ failure assessment; ScvO_2_, central venous oxygen saturation; PBW, predicted body weight; PEEP, positive end-expiratory pressure; ICU, intensive care unit.

### Hemodynamic effects of volume expansion

**Figure 2:**
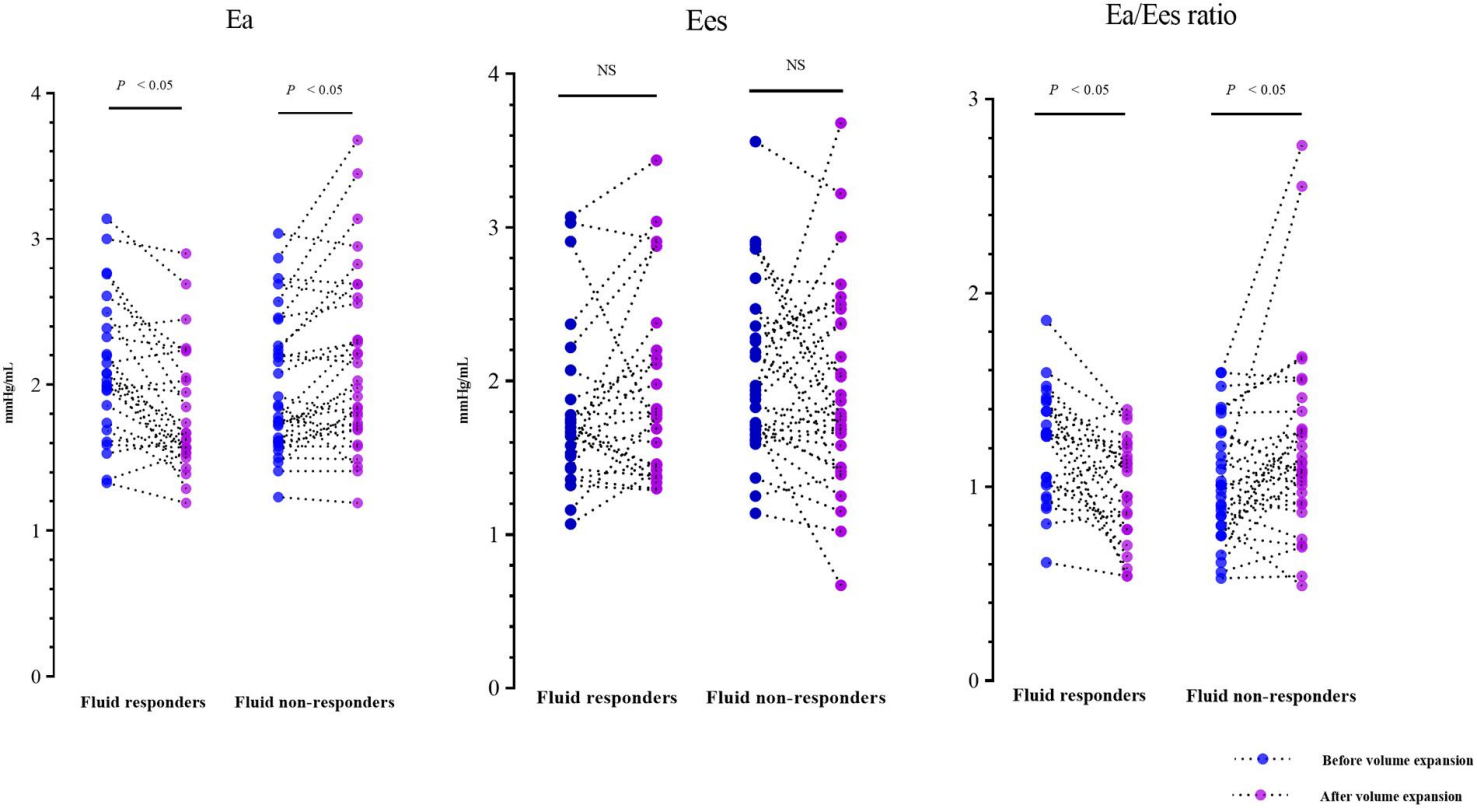
Individual changes in Ea, Ees, and Ea/Ees ratio induced by volume expansion. Ea, effective arterial elastance; Ees, ventricular end-systolic elastance.

The fluid-induced hemodynamic changes are detailed in Table 2. Before VE, most hemodynamic variables showed no significant difference between fluid responders and non-responders. However, fluid responders had a lower CVP and a higher baseline Ea/Ees ratio compared to non-responders. In fluid responders, VE caused notable increases in CVP, arterial pressure, cardiac index, SVI, LVEF, and C_A_, along with significant decreases in SVRI and T_pre−e_/T_tot−s_ ratio; HR remained unchanged. In fluid non-responders, VE significantly increased CVP and arterial pressure, and decreased C_A_, but did not alter HR, cardiac index, SVI, LVEF, T_pre−e_/T_tot−s_ ratio, or SVRI. In fluid responders, VE reduced Ea, while Ees remained stable, leading to a decreased Ea/Ees ratio, indicating VAC optimization. Conversely, in non-responders, VE increased Ea without changing Ees, resulting in an increased Ea/Ees ratio (Figure 2).

**Table 2:** Hemodynamic variables before and after volume expansion.

Variables	Fluid responders (*n* = 26)		Fluid non-responders (*n* = 32)		*P*-value^a^	*P*-value^b^
	Before	After	Before	After		
HR (beats/min)	91 ± 21	92 ± 18	101 ± 22	100 ± 21	0.078	0.139
CVP (mmHg)	9 ± 3	11 ± 3*	11 ± 3	14 ± 4*	0.029	0.009
SAP (mmHg)	107 ± 15	117 ± 18*	103 ± 12	114 ± 14*	0.262	0.556
DAP (mmHg)	55 ± 7	63 ± 10*	57 ± 8	58 ± 8	0.362	0.027
MAP (mmHg)	71 ± 8	81 ± 11*	72 ± 7	76 ± 7*	0.503	0.029
SVI (mL/m^2^)	30.0 ± 5.9	38.0 ± 7.6*	29.8 ± 8.2	30.7 ± 8.9	0.892	0.002
Cardiac index (L/min/m^2^)	2.7 ± 0.7	3.5 ± 0.9*	3.0 ± 1.1	3.0 ± 1.1	0.309	0.095
LVEF (%)	54 ± 10	57 ± 8*	55 ± 10	55 ± 9	0.899	0.331
T_pre-e_/T_tot-s_ ratio (%)	22.7 ± 3.8	20.0 ± 5.4*	20.7 ± 6.1	22.5 ± 7.3	0.142	0.167
SVRI (mmHg/L/m^2^)	24.5 ± 7.6	21.5 ± 6.4*	22.2 ± 5.9	22.4 ± 6.1	0.193	0.592
C_A_ (mL/mmHg)	0.97 ± 0.32	1.25 ± 0.56*	1.14 ± 0.36	0.96 ± 0.35*	0.065	0.020
Ea (mmHg/mL)	2.11 ± 0.48	1.81 ± 0.43*	1.97 ± 0.47	2.15 ± 0.61*	0.276	0.020
Ees (mmHg/mL)	1.80 ± 0.53	1.95 ± 0.62	2.03 ± 0.54	1.95 ± 0.65	0.103	0.991
Ea/Ees ratio	1.22 ± 0.28	0.99 ± 0.26*	1.02 ± 0.30	1.21 ± 0.48*	0.011	0.043

Data were presented as mean ± standard deviation or median with interquartile range.

HR, heart rate; CVP, central venous pressure; SAP, systolic arterial pressure; DAP, diastolic arterial pressure; MAP, mean arterial pressure; SVI, indexed stroke volume; LVEF, left ventricular ejection fraction; SVRI, systemic vascular resistance index; C_A_, arterial compliance; T_pre−e_/T_tot−s_, ratio the ratio of the pre-ejection period to the total systolic period; Ea, effective arterial elastance; Ees effective end-systolic elastance.

a *P*-value for the comparison between fluid responders and non-responders before volume expansion.

b *P*-value for the comparison between fluid responders and non-responders after volume expansion.

* *P* < 0.05 for the intra-group comparison before vs. after volume expansion.

### Relationship between left VAC and fluid responsiveness

Pearson correlation analyses showed that the fluid-induced change in cardiac index was remarkably correlated with the baseline Ea/Ees ratio (*r* = 0.373, *P* = 0.004), but did not correlate with CVP, SVI, Ea, or Ees at baseline (all *P* > 0.05). In addition, the changes in Ea were significantly correlated with the changes in SVI (*r* = −0.810, *P* < 0.001), C_A_ (*r* = −0.741, *P* < 0.001), and SVRI (*r* = 0.406, *P* = 0.002), but did not correlate with the changes in HR (*P* > 0.05).

The univariate logistic regression identified 5 variables potentially associated with fluid responsiveness (all *P* < 0.1) (Table 3), and no potential multicollinearity existed among these variables as all VIF values were less than 2. The abovementioned 5 variables were finally included in the multivariate model, and the multivariable regression analysis indicated that the baseline Ea/Ees ratio was independently associated with fluid responsiveness after adjusting for other confounders (*P* = 0.009) (Table 3). The baseline Ea/Ees ratio exhibited a moderate diagnostic accuracy for identifying fluid responsiveness (AUC of 0.691, 95% CI: 0.555–0.805, *P* = 0.007), with a cutoff value of 1.21, yielding a sensitivity of 61.5% (95% CI: 40.6%–79.8%), a specificity of 75.0% (95% CI: 56.6%–88.5%).

**Table 3:** Logistic regression analyses to identify variables associated with fluid responsiveness.

Variables	Univariable		Multivariable	
	Odds ratio (95% CI)	*p*-value	Odds ratio (95% CI)	*P*-value
Gender (male/female)	0.714 (0.246–2.075)	0.536		
Age (per 1 year increase)	0.996 (0.961–1.033)	0.845		
Shock etiologies (distributive shock as the reference)	1			
Hypovolemic shock	1.733 (0.348–8.641)	0.502		
Cardiogenic shock	0.864 (0.132–5.690)	0.882		
APACHE II score (per 1 point increase)	0.934 (0.849–1.029)	0.166		
SOFA score (per 1 point increase)	**0.768 (0.612–0.964)**	**0.023**		
Arterial lactate (per 1 mmol/L increase)	1.158 (0.874–1.535)	0.306		
ScvO_2_ (per 1% increase)	0.986 (0.951–1.023)	0.447		
Dose of norepinephrine (per 1 μg/kg/min increase)	0.392 (0.025–6.179)	0.505		
Inotropic agents (Yes/No)	1.179 (0.390–3.566)	0.771		
HR (per 1 beats/min increase)	**0.977 (0.952–1.003)**	**0.082**		
CVP (per 1 mmHg increase)	**0.830 (0.698–0.988)**	**0.036**	**0.804 (0.660–0.980)**	**0.031**
MAP (per 1 mmHg increase)	0.975 (0.907–1.049)	0.497		
SVI (per 1 mL/m^2^ increase)	1.005 (0.935–1.081)	0.889		
LVEF (per 1% increase)	0.996 (0.944–1.052)	0.897		
T_pre-e_/T_tot-s_ ratio (per 1% increase)	1.082 (0.973–1.202)	0.145		
SVRI (per 1mmHg/L/m^2^ increase)	1.005 (0.973–1.144)	0.195		
C_A_ (per 0.1 mL/mmHg increase)	**0.854 (0.720–1.014)**	**0.072**		
Ea (per 1 mmHg/mL increase)	1.874 (0.610–5.756)	0.272		
Ees (per 1 mmHg/mL increase)	0.418 (0.144–1.215)	0.109		
Left VAC (Ea/Ees ratio) (per 0.1 increase)	**1.273 (1.048–1.547)**	**0.015**	**1.339 (1.075–1.668)**	**0.009**

Bold indicates *P*-value <0.1. All variables with a *P*-value <0.1 identified in the univariate model are included in the multivariate model.

CI, confidence interval; APACHE, acute physiology and chronic health evaluation; SOFA, sequential organ failure assessment; ScvO_2_, central venous oxygen saturation; HR heart rate; CVP, central venous pressure; MAP, mean arterial pressure; SVI, indexed stroke volume; LVEF, left ventricular ejection fraction; SVRI, systemic vascular resistance index; C_A_, arterial compliance; T_pre−e_/T_tot−s_, ratio the ratio of the pre-ejection period to the total systolic period; Ea, effective arterial elastance; Ees, effective end-systolic elastance; VAC, ventricular-arterial coupling.

## Discussion

The principal findings of this prospective observational study are as follows: 1) preload-dependent patients manifested an uncoupled ventricular-arterial interaction before VE, with a high Ea relative to the Ees, and the baseline left VAC (i.e., Ea/Ees ratio) was independently associated with fluid responsiveness in patients with circulatory shock; 2) VE improved cardiac index in preload-dependent patients, and thus optimized the left VAC in this cohort patients, which was primarily attributed to a reduced arterial load.

From a physiological perspective, arterial load represents the net external opposing forces that hinder ventricular ejection, including various arterial properties such as SVRI, C_A_, arterial impedance, and wave reflection (16). As an integrated measure of cardiac afterload, Ea incorporates the main features of arterial load, encompassing resistive and pulsatile components (16, 17). In our study, VE caused significant decreases in SVRI and Ea, alongside a notable increase in C_A_ among fluid responders, indicating a reduction in arterial load. The reduction of arterial load may be due to decreased sympathetic activity caused by the restoration of blood volume through VE. Conversely, VE increased Ea and decreased C_A_ in fluid non-responders, which is expected because sympathetic stress and the renin-angiotensin system are also activated in cases of fluid overload and congestion induced by further fluid infusion, leading to an increased arterial load. Consistent with this, a previous study on septic shock patients observed a decrease in Ea and systemic vascular resistance, along with an increase in C_A_, after fluid administration in preload responders (18). The possible physiological mechanisms behind the reduced arterial load induced by VE include: first, fluid administration may suppress baroreflex-mediated vasoconstriction caused by hypovolemia in preload responders; second, the effective diameter of the arterial system might expand due to recruitment of collapsed vessels following VE (18); and third, increases in blood flow caused by VE might promote vascular relaxation by increasing nitric oxide production and endothelial shear stress stimulus (19).

The current study replicated the findings of a previous study that VE improved left VAC by decreasing Ea without altering Ees (11), and also revealed the relevance of left VAC and fluid responsiveness in the general population of circulatory shock. Inconsistently, the previous study found that the baseline left VAC predicted fluid responsiveness with an excellent diagnostic accuracy (AUC of 0.84) (11). However, the diagnostic accuracy of left VAC for fluid responsiveness is not good in our study. This difference might partly be due to the different populations studied. Our study enrolled a mixed population of circulatory shock, while the previous study focused on post-cardiac surgical patients, who are often affected by cardiac dysfunction and are more likely to experience cardiogenic shock (20). Compared with our study, fluid responders in the previous study had a lower LVEF (48% vs. 54%), a higher SVRI (47 vs. 24.5 mmHg/L/m^2^), and a higher Ea (2.5 vs. 2.11 mmHg/mL) (11). Therefore, insufficient blood volume could only partially explain the increased baseline arterial load in fluid responders in their study. The release of catecholamines and sympathetic system activation due to postoperative cardiac dysfunction could also contribute to the increased arterial load (21). The combination of insufficient blood volume and postoperative cardiac dysfunction might further influence arterial load and lead to an overestimation of the diagnostic accuracy of left VAC for fluid responsiveness. Additionally, the small sample size (30 patients) in their study might overestimate the effect size and could also contribute to the discrepancy.

Based on these findings, we propose a potential theoretical assumption that VE-induced left VAC optimization in preload-dependent patients may be accomplished primarily by the positive response to VE (i.e., CO increases) to regulate arterial load. In this theoretical scenario, fluid loading causes varied changes in CO and cardiac afterload in patients with different volume states, thereby affecting the ventricular-arterial interactions, which in turn affect ventricular ejection. This may explain why the LVEF improved in fluid responders but remained unchanged in non-responders in our study. Notably, numerous studies have also indicated a link between left VAC optimization and cardiovascular responses to hemodynamic interventions (22–24). The normalization of left VAC was reported to be associated with SV responsiveness to norepinephrine infusion (22, 23), and abnormal left VAC effectively predicted oxygen consumption responses to hemodynamic interventions (24). Furthermore, our recent study demonstrated the beneficial effects of left VAC optimization on lactate clearance and clinical prognosis in septic shock (4, 8). The above findings underline the significance of left VAC in understanding the cardiovascular physiological responses to hemodynamic therapy and suggest the possibility of left VAC as a potential therapeutic target.

Our study had several limitations. First, the etiology of shock may represent a primary source of heterogeneity. However, it would not diminish the reliability of the VAC concept in understanding cardiovascular physiology related to VE, because the shock etiology was not independently associated with fluid responsiveness. Second, concerns may arise about the potential impacts of invasive mechanical ventilation, vasopressors, and inotropes on the results. However, these impacts, if any, would be minimal because their parameters and dosages remained unchanged throughout the study period. Third, there is a lack of assessment of right ventricular function during the study period, which may limit our comprehensive understanding of VE-related cardiovascular physiology. Furthermore, arterial load was assessed using the two-element Windkessel model (i.e., SVRI and C_A_) and an integrative simplification (i.e., Ea). Simplified estimations of arterial load based on classical formulas may be insufficient to accurately reflect true arterial load due to the mathematical coupling effect, which could result in a potential bias in the findings. Although an integrated evaluation of arterial impedance and wave reflection may provide more precise information on arterial load, measuring and interpreting these variables at the bedside remains challenging. Lastly, there are criticisms regarding the estimation of ESP based on radial arterial pressure. Indeed, the gold standard for measuring ESP typically requires an invasive ventricular pressure catheter, and the radial artery signal may not be optimal for estimating ESP due to the phenomenon of pulse wave amplification, which refers to the physiological increase in systolic pressure from the aorta to the periphery (25). Therefore, the noninvasive estimation of Ea and Ees using echocardiography may deviate from the true values, thereby causing potential bias in the results. Despite this, the estimation of the Ea/Ees ratio would not be affected because of similar influences on the calculations of Ea and Ees.

## Conclusion

In mechanically ventilated patients with circulatory shock, VE improved the left VAC in preload-dependent patients, primarily by reducing arterial load. Left VAC was independently associated with fluid responsiveness. Left VAC has specific advantages in understanding the cardiovascular physiology associated with VE.

## Data Availability

The original contributions presented in the study are included in the article/Supplementary Material, further inquiries can be directed to the corresponding authors.
