# Supplementary material for: Hemodynamic effects of volume expansion on left ventricular-arterial interactions in circulatory shock—a prospective observational study
Source: Front Cardiovasc Med. 2026 Jan 27;13:1726410. doi: 10.3389/fcvm.2026.1726410 (PMC12886503; doi:10.3389/fcvm.2026.1726410)
Supplement: Supplementary file 1 [file Table1.docx]

Table S1. Intra-observer reproducibility for directly measured ultrasound variables

| Variables | LVEDV | LVESV | VTI | LVEF | T_pre-e_ | T_tot-s_ |
| --- | --- | --- | --- | --- | --- | --- |
| Coefficient of variation (%) | 3.2 (2.6 – 3.8) | 6.3 (4.5 – 8.1) | 3.2 (1.4 – 4.9) | 4.9 (3.1 – 6.8) | 5.1 (3.4 – 6.9) | 1.7 (1.2 - 2.2) |
| Least significant change (%) | 5.1 (4.1 - 6.0) | 10.1 (7.2 – 12.9) | 5.1 (2.2 – 7.9) | 7.9 (5.0 – 10.8) | 8.2 (5.4 - 11.0) | 2.7 (1.9 - 3.5) |

Data was presented as mean value and its corresponding 95% confidence intervals.

LVEDV left ventricular end-diastolic volume; LVESV left ventricular end-systolic volume; VTI aortic velocity-time integral; LVEF left ventricular ejection fraction; T_pre-e_ pre-ejection time; T_tot-s_ total systolic time.
